# Visual and Anatomical Outcomes of Vitrectomy with ERM and ILM Peeling in Idiopathic and Secondary ERM

**DOI:** 10.3390/diagnostics16193175

**Published:** 2026-09-30

**Authors:** İsa Yuvaci, Kübra Özata Gündoğdu, Eda Gümrükçüoğlu

**Affiliations:** 1 Department of Ophthalmology, Faculty of Medicine, Sakarya University, 54187 Sakarya, Turkey; 2 Department of Ophthalmology, Sakarya Training and Research Hospital, 54100 Sakarya, Turkey; kubraoz78@hotmail.com (K.Ö.G.); gumrukcuoglueda@gmail.com (E.G.)

**Keywords:** epiretinal membrane, pars plana vitrectomy, optical coherence tomography, central macular thickness

## Abstract

**Background/Objectives:** To evaluate the visual and anatomical outcomes of pars plana vitrectomy (PPV) with epiretinal membrane (ERM) and internal limiting membrane (ILM) peeling and to assess the impact of etiological differences on postoperative outcomes. **Methods:** This retrospective study included 57 eyes of 51 patients who underwent PPV for ERM, including 34 eyes with idiopathic ERM and 23 eyes with secondary ERM. Patients were classified as having idiopathic or secondary ERM based on clinical history, fundus examination, and optical coherence tomography (OCT) findings. Best-corrected visual acuity (BCVA), central macular thickness (CMT), foveal contour, intraoperative findings, and postoperative outcomes at 1, 3, 6, and 12 months were analyzed. Factors associated with final visual acuity were evaluated using correlation analyses and multivariate linear regression analysis. **Results:** The mean age of patients was 71.43 ± 6.46 years. BCVA improved significantly from 0.78 ± 0.35 logMAR preoperatively to 0.62 ± 0.44 logMAR at 12 months postoperatively (*p* = 0.004). Mean CMT decreased significantly from 411.68 ± 124.13 µm to 243.03 ± 89.25 µm at 12 months (*p* < 0.001). Improvement in foveal contour was observed in 70.2% of eyes; however, this anatomical improvement was not associated with better visual outcomes. In the multivariable analysis, preoperative BCVA was significantly associated with 12-month BCVA (*p* = 0.038), although the overall regression model did not reach statistical significance (*p* = 0.134). Although changes in BCVA and CMT did not differ significantly between idiopathic and secondary ERM groups, baseline BCVA-adjusted analysis showed significantly worse final BCVA in the secondary ERM group. **Conclusions:** ERM surgery with ILM peeling resulted in significant improvements in both visual and anatomical outcomes, supporting its effectiveness in achieving functional and anatomical improvement.

## 1. Introduction

Epiretinal membrane (ERM) is a vitreomacular interface disease that develops as a result of fibroglial proliferation on the inner surface of the retina. Cases with etiologies that cause inflammation, such as trauma, ocular surgery, and retinal vascular diseases, are classified as secondary ERM, whereas cases in which no etiological factor can be identified constitute the idiopathic ERM group [1]. Although the majority of ERM cases are considered idiopathic, it has been reported that they are most commonly seen in individuals aged 50 years and older [2].

Most patients with ERM are asymptomatic and have extrafoveal membranes. However, when the ERM involves the macular or perimacular region, associated retinal traction and edema may adversely affect visual acuity. Common symptoms include decreased visual acuity, blurred vision, metamorphopsia, impaired stereopsis, and aniseikonia [3].

Optical coherence tomography (OCT) is an objective, cross-sectional, and noninvasive method used for evaluating the retinal surface. In patients with ERM, OCT can be used to assess the epiretinal membrane, central macular thickness, foveal contour, retinal edema, and traction-related serous retinal detachment [4].

Medical treatment has no direct role in ERM management. However, anti-VEGF agents, steroids, or nonsteroidal anti-inflammatory drugs may be used to treat macular edema associated with conditions causing secondary ERM, such as diabetic retinopathy, retinal vascular occlusion, and uveitis [3]. The main treatment option for ERM is surgery. The primary indications for ERM surgery are decreased visual acuity or symptomatic metamorphopsia [4,5].

Although visual and anatomical improvement after ERM surgery is well established, the relationship between anatomical and functional recovery remains variable. Moreover, most studies have focused on idiopathic ERM, with limited comparative data on secondary ERM. Therefore, the aim of this study was to evaluate visual and anatomical changes following ERM surgery, compare surgical outcomes between idiopathic and secondary ERM, and investigate the relationship between anatomical recovery and postoperative visual outcomes.

## 2. Materials and Methods

Medical records of patients who underwent pars plana vitrectomy (PPV) for the diagnosis of ERM at the Department of Ophthalmology, Sakarya University Training and Research Hospital between 2019 and 2023 were retrospectively reviewed. The study was conducted in accordance with the principles of the Declaration of Helsinki. Ethical approval was obtained from the local ethics committee (approval number: E-71522473-050.01.04-300119-320). Written informed consent was obtained from all participants.

A total of 57 eyes of 51 patients who underwent surgery for ERM and had at least one year of regular follow-up were included in the study. Six patients underwent bilateral surgery. Clinical findings were analyzed preoperatively and at postoperative months 1, 3, 6, and 12.

ERM cases were classified as idiopathic or secondary based on the presence of an associated retinal disease. ERMs associated with diabetic retinopathy (DR), age-related macular degeneration (AMD), or retinal vein occlusion (RVO) were classified as secondary. All diabetic cases had non-proliferative diabetic retinopathy (NPDR), and none had fibrovascular membrane formation or tractional retinal detachment. However, due to the retrospective nature of the study, the temporal relationship between the associated retinal disease and ERM development could not be established in all cases.

Patients with a follow-up period shorter than 12 months; incomplete surgical or follow-up data; a history of previous retinal surgery, lamellar macular hole, full-thickness macular hole, neovascular age-related macular degeneration, geographic atrophy involving the fovea, other retinal diseases significantly affecting central vision (such as retinal dystrophies), and ERM secondary to trauma were excluded from the study.

From the patient records, demographic characteristics (age and sex), ocular and systemic medical history, best-corrected visual acuity (BCVA), intraocular pressure (IOP), lens status (phakic or pseudophakic), and OCT findings were collected. Surgical variables, including dye application time, duration of surgery, tamponade agent, intraoperative complications, and laser application, were also recorded, together with postoperative follow-up duration.

BCVA was measured using the Snellen chart and converted to logarithm of the minimum angle of resolution (logMAR) values. Fundus examinations were performed using a +90 diopter lens. OCT imaging was carried out using the Cirrus HD-OCT 400 system (Zeiss Meditech Inc., Dublin, CA, USA). Preoperatively, central macular thickness (CMT) as well as the presence of a pseudomacular hole, vitreomacular traction (VMT), and subretinal fluid were evaluated. During postoperative follow-up, CMT and structural changes in the retinal surface were recorded. All OCT images were evaluated by a single ophthalmologist using predefined criteria. The observer was not masked to the clinical data.

**Figure 1:**
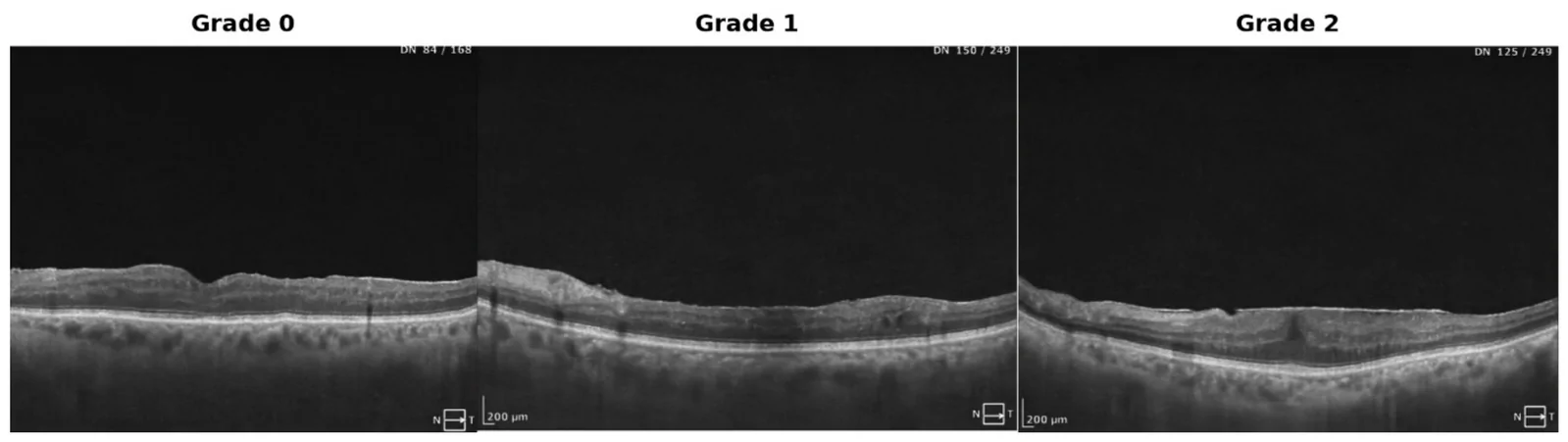
Representative optical coherence tomography (OCT) images illustrating foveal contour grading: Grade 0, presence of a normal foveal depression; Grade 1, flat foveal contour without a foveal depression; and Grade 2, elevated foveal contour.

The foveal contour was graded based on OCT findings as Grade 0 (presence of a foveal depression), Grade 1 (flat fovea), or Grade 2 (elevated fovea) (Figure 1) [6]. The study included eyes with preoperative Grade 1 or Grade 2 foveal contour. Foveal contour improvement was defined as a postoperative reduction from Grade 1 to Grade 0 or from Grade 2 to Grade 1 or Grade 0. ERM recurrence was defined as the reappearance of an epiretinal membrane on fundus examination and OCT during postoperative follow-up after its surgical removal.

Macular edema was defined on OCT as the presence of intraretinal cystoid spaces, subretinal fluid, and diffuse retinal thickening. The underlying cause of edema was determined based on the associated retinal disease together with the clinical and OCT findings, including diabetic retinopathy and retinal vein occlusion.

All surgeries were performed by a single experienced vitreoretinal surgeon using the same surgical system (DORC EVA, Zuidland, The Netherlands) with a 25-gauge transconjunctival three-port pars plana vitrectomy (PPV) technique. Core vitrectomy was performed in all eyes, followed by intravitreal triamcinolone injection to assess the status of the posterior vitreous. In eyes without a pre-existing posterior vitreous detachment, posterior vitreous detachment was induced using the vitreous cutter. Dual blue dye was then applied to the posterior pole for 1 min. If adequate visualization was not achieved, additional staining was performed in 30-s intervals until sufficient visualization was obtained. ERM peeling was performed with the aim of completely removing the membrane from the macula and posterior pole without leaving residual tissue that could cause traction. ILM peeling was subsequently performed in the macular region, with particular attention to the foveal area, to remove residual tractional tissue and minimize residual tangential traction. A fixed peeling diameter was not used; the extent of peeling was determined intraoperatively according to the area required to achieve complete removal of the tractional membrane. Air tamponade was routinely used in uncomplicated cases. Sulfur hexafluoride (SF_6_) or perfluoropropane (C_3_F_8_) gas tamponade was used selectively in eyes with technically challenging membrane peeling or when an intraoperative retinal break was identified or suspected. In eyes with cataracts, cataract surgery was performed using the phacoemulsification technique during the same surgical session as PPV. Intraoperative intravitreal anti-VEGF injection was administered in selected eyes with macular edema related to the underlying retinal disease.

### Statistical Analysis

All statistical analyses were performed using IBM SPSS Statistics for Windows, Version 21.0 (IBM Corp., Armonk, NY, USA). Descriptive statistics were expressed as mean ± standard deviation. Data distribution was assessed using the Kolmogorov–Smirnov test for normality. Parametric tests were used for normally distributed variables, while nonparametric tests were used for variables that were not normally distributed. To account for the potential within-subject correlation arising from bilateral cases, a sensitivity analysis was performed in which one eye was randomly selected using a computer-generated randomization procedure for each of the six patients who underwent bilateral surgery. This resulted in a sensitivity dataset comprising 51 eyes from 51 patients. Changes over time were analyzed using repeated-measures ANOVA for normally distributed variables and the Friedman test for non-normally distributed variables. When a significant overall difference was detected, pairwise comparisons were performed with Bonferroni correction for multiple comparisons. Categorical variables were compared using the chi-square test. Comparisons between two groups were performed using the independent-samples t test (or the Mann–Whitney U test), and comparisons among multiple groups were conducted using one-way ANOVA (or the Kruskal–Wallis test). The relationship between continuous variables and final visual acuity was evaluated using Pearson or Spearman correlation analysis. Multivariable linear regression analysis was performed to evaluate factors associated with final visual acuity. Variables included in the multivariable regression model were selected a priori based on their clinical relevance as potential preoperative predictors of postoperative visual outcome. Age, preoperative BCVA, preoperative CMT, and ERM etiology were included to represent baseline demographic, functional, anatomical, and disease-related characteristics, respectively. A *p* value < 0.05 was considered statistically significant.

## 3. Results

### 3.1. Preoperative Characteristics

A total of 57 eyes from 51 patients who underwent PPV for ERM were included in the study. Of the patients, 25 (49%) were male and 26 (51%) were female, with a mean age of 71.43 ± 6.46 years (Table 1). Preoperative BCVA was 0.78 ± 0.35 logMAR, and the mean IOP was 14.66 ± 3.58 mmHg.

Anterior segment examination revealed that 28 eyes (49.1%) were phakic and 29 eyes (50.9%) were pseudophakic. The etiology of ERM was idiopathic in 34 eyes (59.6%) and secondary in 23 eyes (40.4%). Among secondary ERM cases, the underlying etiologies were DR in 17 eyes (29.8%), AMD in 3 eyes (5.3%), and RVO in 3 eyes (5.3%) (Table 1). Preoperatively, 17 eyes (29.8%) had Grade 1 and 40 eyes (70.2%) had Grade 2 foveal contour on OCT. The mean preoperative CMT value was 411.68 ± 124.13 µm. A pseudomacular hole was present in 7 eyes (12.3%), vitreomacular traction (VMT) in 9 eyes (15.8%), and macular edema in 9 eyes (15.8%).

### 3.2. Intraoperative Findings

The mean surgical duration was 26.44 ± 10.90 min, calculated as the interval between the first trocar insertion and the final trocar removal. Retinal staining with Dual Blue dye was performed in all cases, with a mean staining time of 141.56 ± 49.37 s. Combined PPV and phacoemulsification surgery was performed in 10 eyes (17.5%).

Postoperatively, air tamponade was used in 48 eyes (84.2%), C_3_F_8_ gas in 7 eyes (12.3%), and SF_6_ gas in 2 eyes (3.5%). Intravitreal anti-VEGF injection was administered during PPV in 9 eyes (17.6%).

### 3.3. Postoperative Outcomes

**Figure 2:**
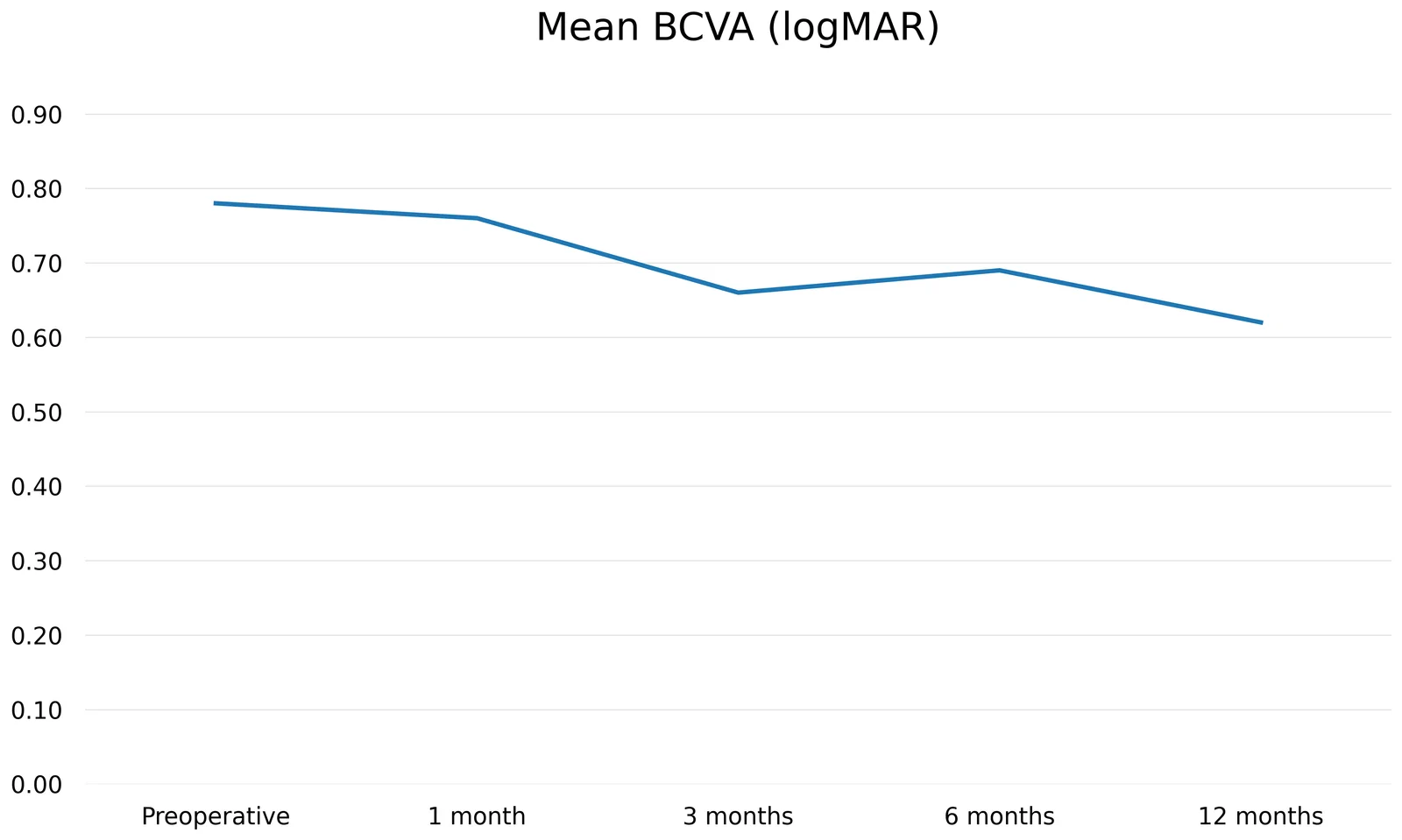
Changes in best-corrected visual acuity (BCVA) over the 12-month postoperative follow-up period.

Mean BCVA was 0.78 ± 0.35 logMAR preoperatively and 0.76 ± 0.46 logMAR at 1 month, 0.66 ± 0.43 logMAR at 3 months, 0.69 ± 0.52 logMAR at 6 months, and 0.62 ± 0.44 logMAR at 12 months postoperatively (Figure 2). The mean change in BCVA from baseline to 12 months was −0.16 ± 0.46 logMAR. Comparisons between preoperative BCVA and postoperative BCVA demonstrated significant improvement at 3, 6, and 12 months, while the improvement at 1 month was not statistically significant (Table 2). Pairwise comparisons of postoperative BCVA showed significant improvement between 1 and 3 months (*p* = 0.001), 1 and 6 months (*p* = 0.007), and 1 and 12 months (*p* < 0.001). No significant differences were observed between 3 and 6 months (*p* = 1.000), 3 and 12 months (*p* = 0.402), or 6 and 12 months (*p* = 0.132).

Mean IOP was 14.66 ± 3.58 mmHg preoperatively and 15.14 ± 3.34, 14.74 ± 3.33, 15.10 ± 3.48, and 16.50 ± 8.36 mmHg at postoperative months 1, 3, 6, and 12, respectively. None of the postoperative IOP measurements differed significantly from the preoperative value (all *p* > 0.05). Transient hypotony (IOP < 6 mmHg) was observed in 3 eyes (5.3%) within the first postoperative week and resolved spontaneously.

Correlation analysis revealed no significant association between final BCVA and preoperative CMT, staining duration, surgical time, or age (all *p* > 0.05). However, a significant positive correlation was found between preoperative BCVA and final BCVA (*p* = 0.007, r = 0.351).

**Figure 3:**
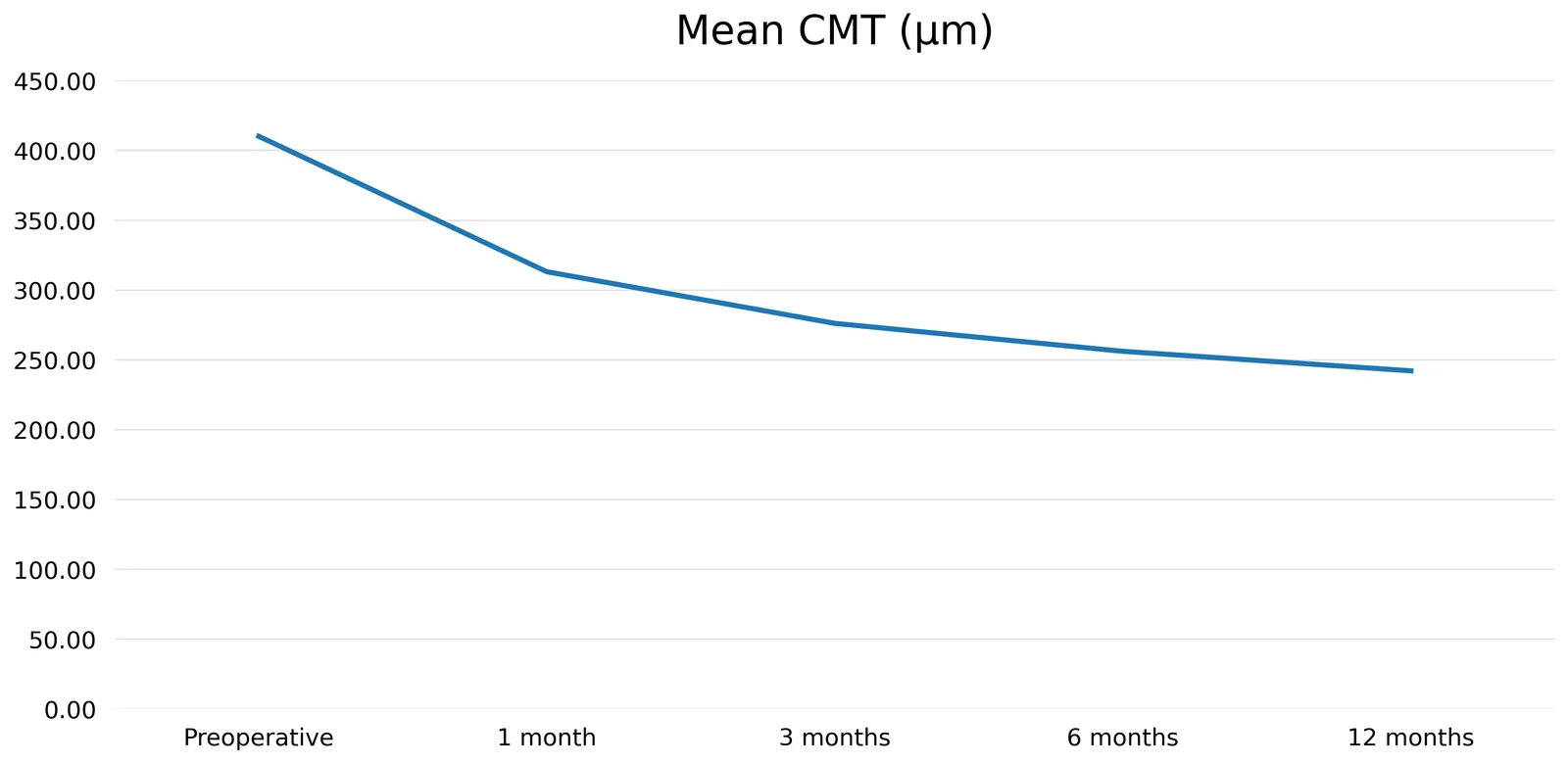
Changes in central macular thickness (CMT) over the 12-month postoperative follow-up period.

Mean CMT decreased from 411.68 ± 124.13 µm preoperatively to 313.24 ± 127.94, 275.63 ± 107.70, 256.71 ± 100.23, and 243.03 ± 89.25 µm at postoperative months 1, 3, 6, and 12, respectively (Figure 3). CMT was significantly lower than the preoperative value at all postoperative visits (all *p* < 0.001) (Table 3), with an overall mean reduction of 168.64 ± 119.65 µm at 12 months. A significant correlation was also observed between preoperative and 12-month CMT values (*p* = 0.002, r = 0.409).

No significant correlation was observed between the change in CMT and the change in BCVA from baseline to 12 months (r = 0.081, *p* = 0.665).

**Figure 4:**
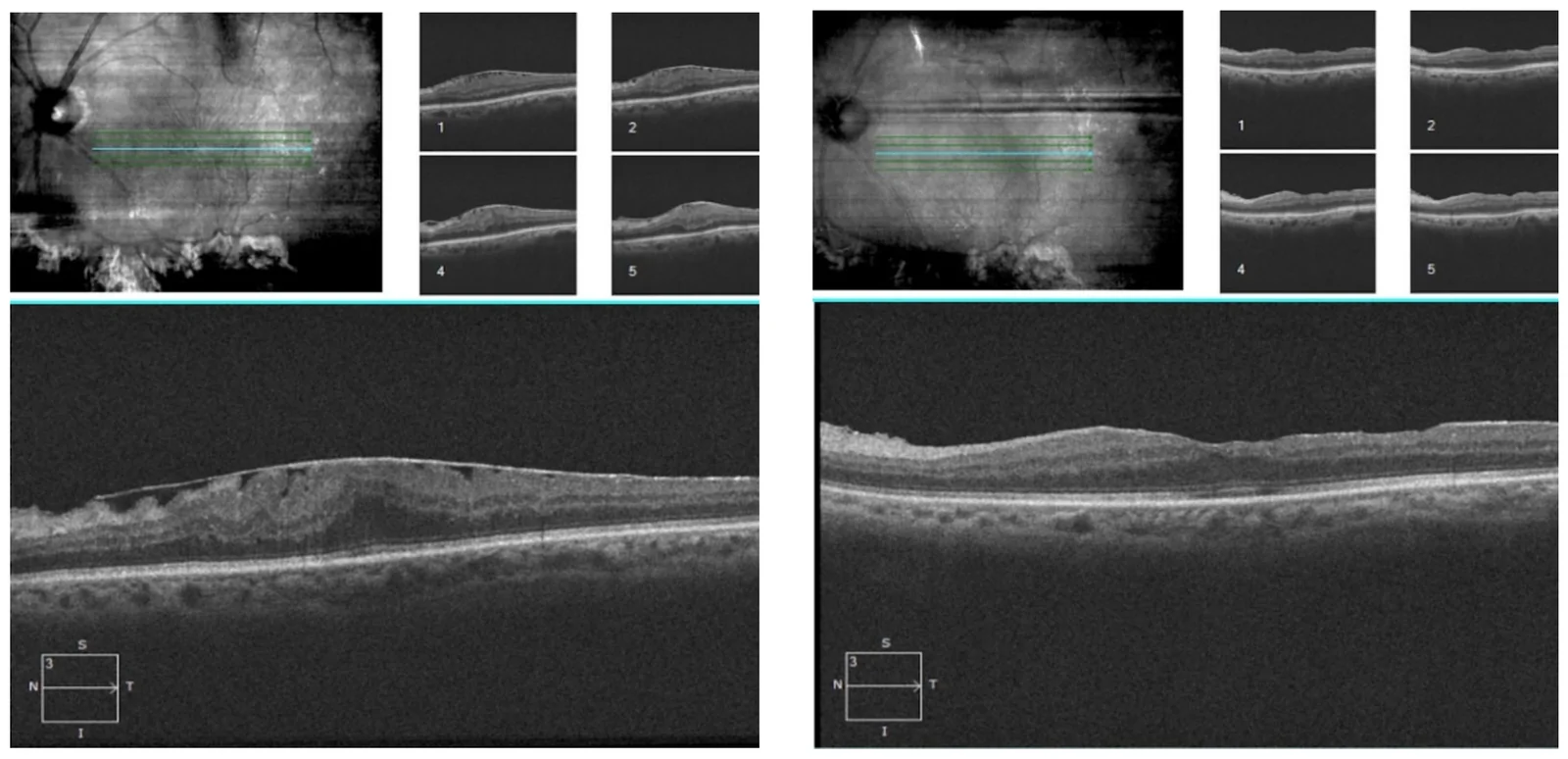
Representative preoperative and 12-month postoperative optical coherence tomography (OCT) images of an eye with an epiretinal membrane, demonstrating postoperative anatomical improvement with reduction in retinal thickness and restoration of the foveal contour.

Postoperative OCT demonstrated improvement in foveal contour in 40 eyes (70.2%). Specifically, regression from Grade 2 to Grade 0 was observed in 20 eyes (35.1%), from Grade 2 to Grade 1 in 8 eyes (14.0%), and from Grade 1 to Grade 0 in 12 eyes (21.1%). At 12 months, 32 eyes (56.1%) had a Grade 0 foveal contour. Although eyes with a Grade 0 foveal contour at 12 months had significantly lower CMT than those with Grade 1 or 2 contours (*p* < 0.001), BCVA did not differ significantly between the groups (*p* = 0.477). Representative preoperative and 12-month postoperative OCT images demonstrating anatomical improvement following ERM surgery are shown in Figure 4.

Nine eyes with ERM secondary to diabetic retinopathy received intraoperative anti-VEGF treatment for concomitant diabetic macular edema. No significant differences were observed between eyes receiving and not receiving intraoperative anti-VEGF treatment in preoperative CMT (*p* = 0.888), final CMT (*p* = 0.239), or CMT change from baseline to the final follow-up (*p* = 0.218). Macular edema was detected on postoperative OCT in 6 eyes (10.5%), resolving spontaneously in 2 eyes (3.5%). Persistent macular edema was treated with two doses of intravitreal bevacizumab in 2 eyes (3.5%), three doses of bevacizumab in one eye, and a single intravitreal dexamethasone implant in one eye. Of these four eyes, one had idiopathic ERM, two had ERM secondary to diabetic retinopathy, and one had ERM secondary to branch retinal vein occlusion. In a sensitivity analysis excluding all 13 eyes that received intraoperative or postoperative pharmacological treatment, CMT remained significantly reduced from baseline at 1, 3, 6, and 12 months (all *p* < 0.001).

Intraoperative retinal tears occurred in 6 eyes (10.5%), and endolaser photocoagulation was applied to all affected eyes. No statistically significant differences were observed between eyes with intraoperative retinal tears (*n* = 6) and those without tears (*n* = 51) in terms of preoperative BCVA, 12-month BCVA, changes in BCVA, or changes in CMT (all *p* > 0.05).

Among the 18 eyes that remained phakic after PPV, cataract progression was observed in 9 eyes (50.0%), all of which subsequently underwent cataract surgery during the follow-up period. The mean interval from PPV to cataract surgery was 9.89 ± 8.45 months. Among these nine eyes, mean BCVA was 0.63 ± 0.38 logMAR before cataract surgery and 0.56 ± 0.32 logMAR after cataract surgery, with no statistically significant difference (*p* = 0.750). Similarly, initial and final BCVA did not differ significantly between these eyes and those that were pseudophakic at baseline (*p* = 0.675, *p* = 0.798, respectively). Additionally, no significant differences were observed between eyes undergoing combined PPV and phacoemulsification (*n* = 10) and those undergoing PPV alone (*n* = 47) in preoperative BCVA (*p* = 0.639), final BCVA (*p* = 0.680), or the change in BCVA from baseline to the final follow-up (*p* = 0.332).

The mean surgical duration was 26.44 ± 10.90 min, and no significant correlation was found between surgical duration and final BCVA (*p* = 0.686). Similarly, no significant correlation was observed between surgical duration and final CMT (*p* = 0.094). Among eyes without cataract progression, mean surgical duration was 25.83 ± 11.55 min, compared with 30.13 ± 9.67 min in eyes with cataract progression (*p* = 0.22).

Baseline BCVA was significantly better in the idiopathic ERM group than in the secondary ERM group (0.69 ± 0.32 vs. 0.91 ± 0.37 logMAR, *p* < 0.001). There was no significant difference in baseline CMT between the idiopathic and secondary ERM groups (406.44 ± 122.62 µm vs. 419.43 ± 128.72 µm, *p* = 0.702). No statistically significant differences were observed between the groups in BCVA recovery or CMT reduction (*p* = 0.107 and *p* = 0.052, respectively). However, after adjustment for baseline BCVA, secondary ERM was associated with significantly worse final BCVA compared with idiopathic ERM (B = 0.341 logMAR, 95% CI: 0.112–0.569, *p* = 0.004). Given the heterogeneity and small subgroup sizes within the secondary ERM group, these etiological comparisons should be considered exploratory.

During follow-up, ERM recurrence was detected in 18 eyes (31.6%) based on fundus examination and OCT findings. The recurrent membranes were thin, without significant retinal traction or clinically relevant symptoms, and none required repeat surgery. Of these, 12 recurrences (35.3%) occurred in the idiopathic ERM group and 6 (26.1%) in the secondary ERM group, with no significant difference in recurrence rates between the groups (*p* = 0.567). Recurrence rates did not differ significantly among the secondary ERM subgroups (*p* = 0.638). Furthermore, no significant differences were observed between eyes with and without recurrence in terms of 12-month BCVA, 12-month CMT, CMT change, or BCVA change (*p* = 0.263, *p* = 0.311, *p* = 0.693, and *p* = 0.635, respectively).

In the multivariable linear regression analysis, preoperative BCVA was significantly associated with 12-month BCVA among the variables included in the model (B = 0.394, β = 0.297, *p* = 0.038). Preoperative CMT (*p* = 0.473), age (*p* = 0.483), and ERM etiology (*p* = 0.770) were not significantly associated with 12-month BCVA. The model explained 13.4% of the variance in 12-month BCVA (R^2^ = 0.134, adjusted R^2^ = 0.062), although the overall model did not reach statistical significance (F(4,48) = 1.853, *p* = 0.134).

A sensitivity analysis restricted to one eye per patient (51 eyes from 51 patients) yielded findings consistent with the primary analysis. BCVA improved from 0.79 ± 0.35 logMAR preoperatively to 0.66 ± 0.45 logMAR at final follow-up (mean change: −0.13 ± 0.49 logMAR, *p* = 0.039). CMT decreased from 403.22 ± 124.58 µm preoperatively to 243.49 ± 95.27 µm at final follow-up (mean change: −159.73 ± 120.41 µm, *p* < 0.001). These findings indicate that restricting the analysis to one eye per patient did not materially alter the principal visual and anatomical outcomes (Table 4).

## 4. Discussion

In this study, we evaluated the functional and anatomical outcomes following PPV with membrane peeling for ERM. Consistent with previous studies, our findings showed a significant improvement in BCVA and a marked reduction in CMT after surgery.

Visual recovery after ERM surgery may continue for several months. In our study, BCVA did not improve significantly in the first postoperative month but showed significant improvement by the third month. No significant differences were observed among the 3-, 6-, and 12-month measurements, suggesting that visual recovery became evident by the third postoperative month and remained relatively stable thereafter. Similarly, Minami et al., in a study of 114 eyes with idiopathic ERM, reported that BCVA improved at one month and continued to improve until three months after surgery [6]. Kwon et al. also demonstrated a progressive increase in the proportion of eyes achieving visual improvement between one and seven months postoperatively [7]. Another study reported a mean time of 4.82 months to achieve the best postoperative BCVA [8]. When these findings are considered together, it is understood that improvement in vision after ERM surgery occurs gradually in the early postoperative period, but this process may vary among patients.

Contraction of myofibroblasts on the retinal surface may lead to progressive retinal wrinkling and distortion over time, and traction and edema caused by ERM can result in the loss of the foveal depression. Even after successful surgical removal of ERM via vitreoretinal surgery, restoration of the original retinal contour and reformation of the foveal pit remain limited in many cases [4]. Saraceno et al. reported that changes in foveal contour were not correlated with visual acuity, and that only 20% of patients demonstrated restoration of normal foveal configuration. Moreover, they noted that some patients showed significant visual improvement despite the absence of postoperative improvement in foveal contour [9].

Mathew et al. reported restoration of foveal depression in foveal contour in 17.1% of eyes following idiopathic ERM surgery. Although retinal thickness decreased significantly in eyes showing greater restoration of foveal contour, this anatomical improvement was not accompanied by a significant improvement in visual acuity [10]. In our study, foveal contour improved in 40 eyes (70.2%) at 12 months. Eyes with a Grade 0 foveal contour had better anatomical outcomes than those with Grade 1–2 contours, but BCVA did not differ significantly between the groups. Similarly, another study involving 43 patients who underwent surgery for idiopathic ERM reported postoperative foveal pit restoration in 72% of cases; however, no significant differences in BCVA or foveal avascular zone (FAZ) values were found between patients with and without foveal pit restoration at 12 months [11]. In a nationwide multicenter retrospective study conducted across 22 academic centers in Turkey involving 634 eyes, restoration of normal or shallow foveal contour was reported in 54% of eyes following surgery. Functional improvement was emphasized to be more likely in patients with better baseline BCVA and an intact ellipsoid zone (EZ) [12].

Kwon et al. reported that final BCVA was significantly correlated with preoperative BCVA, preoperative foveal thickness, and final foveal thickness, suggesting that preoperative BCVA and foveal thickness may be useful predictors of postoperative visual outcomes [7]. Mean foveal thickness decreased from 409.7 ± 107.9 µm preoperatively to 288.6 ± 66.1 µm at seven months after surgery. A systematic review analyzing 19 studies on idiopathic ERM reported that preoperative BCVA was the only variable consistently associated with postoperative BCVA, while preoperative CMT was not correlated with visual outcomes [13]. In agreement with this review, preoperative CMT was not significantly associated with 12-month BCVA in our study, whereas preoperative BCVA showed a significant association with 12-month BCVA in the multivariable analysis. However, this finding should be interpreted cautiously, as the overall regression model did not reach statistical significance.

Recurrence following surgical removal of ERM is considered an uncommon complication, and repeat surgical intervention is typically reserved for a limited number of cases. ERM recurrence has been associated with the absence of simultaneous internal limiting membrane (ILM) peeling, the presence of ERM in the fellow eye, or poor preoperative visual acuity [14]. In our study, ERM recurrence was observed in 18 eyes (31.6%), none of which required repeat surgery during follow-up. Recurrence rates were 35.3% in the idiopathic ERM group and 26.1% in the secondary ERM group, with no significant difference between groups (*p* = 0.567). Sandali et al. reported that recurrences were thin and asymptomatic in all cases where ILM peeling had been performed, attributing this to the elimination of the scaffold required for myofibroblast proliferation. They further suggested that idiopathic ERMs exhibit greater proliferative potential and recommended systematic ILM peeling to reduce recurrence risk [14]. Kang et al. reported a higher one-year recurrence rate in secondary ERM compared with idiopathic ERM (20% vs. 4.8%) [15]. Another OCT-based study evaluating 301 eyes reported a recurrence rate of 39.5% following idiopathic ERM surgery [16]. Additionally, a single-center study with a five-year follow-up reported a recurrence rate of 58%, highlighting the potential for high recurrence rates with long-term follow-up [17]. Although the recurrence rate in our study appears relatively high, it is consistent with rates reported in previous studies using OCT to evaluate anatomical recurrence. Moreover, recurrence was not associated with poorer visual or anatomical outcomes. This finding suggests that some recurrent membranes detected on OCT may represent subtle anatomical changes without sufficient functional significance to adversely affect visual outcomes during the follow-up period.

Intraoperative complications such as intraretinal hemorrhage, retinal holes or tears, and lens damage in phakic eyes may occur during ERM surgery. In our study, intraoperative iatrogenic retinal tears occurred in 6 eyes (10.5%). Dogramaci et al. reported an incidence of 9.38% in eyes undergoing PPV for macular pucker, while Neuhann et al. reported an incidence of 12.7% in eyes undergoing 23- or 25-gauge vitrectomy with ERM or ILM peeling [18,19]. Thus, although the retinal tear rate in our study may appear relatively high, it is within the range reported in comparable surgical series. Postoperatively, nuclear sclerotic cataract has been reported to develop in 10–70% of phakic eyes [20]. In elderly patients, cataract progression may occur early after surgery; therefore, combined phacoemulsification with intraocular lens implantation may be performed concurrently with ERM surgery in selected cases. In our study, combined vitrectomy, phacoemulsification, and intraocular lens implantation were performed in 10 eyes. Among the remaining 18 phakic eyes, cataract progression occurred in 9 eyes (50.0%) during follow-up, all of which subsequently underwent cataract surgery.

Cheng et al. reported that vitrectomy duration was not associated with the progression of nuclear sclerosis, despite a high incidence of nuclear sclerosis progression at one year [21]. Similarly, in our study, mean surgical duration did not differ significantly between eyes with and without cataract progression (30.13 ± 9.67 vs. 25.83 ± 11.55 min, respectively; *p* = 0.22). Similarly, Falkner et al. reported comparable BCVA outcomes between eyes that underwent cataract extraction in a separate session after ERM surgery and eyes that were pseudophakic at baseline [22]. In agreement with this, we found no significant differences in baseline or final BCVA between eyes that developed cataracts postoperatively and those that were pseudophakic preoperatively.

The comparison between idiopathic and secondary ERM should be interpreted cautiously because the secondary ERM group comprised heterogeneous retinal conditions with potentially different visual prognoses. Moreover, some etiological subgroups included only a small number of eyes. Therefore, these comparisons should be considered exploratory, and the absence of statistically significant differences should not be interpreted as evidence of equivalent postoperative outcomes.

The main limitations of this study include its retrospective design, single-center setting, and relatively small sample size, which may limit the generalizability of the findings. The secondary ERM group was heterogeneous, comprising different underlying pathologies with distinct clinical characteristics and prognoses. Subjective assessments of visual quality, such as vision-related quality-of-life questionnaires, were not included, and the analysis was limited to anatomical and functional parameters. Furthermore, OCT biomarkers that may have prognostic value, such as ellipsoid zone integrity, disorganization of retinal inner layers (DRIL), and ectopic inner foveal layers (EIFL), were not consistently available and therefore could not be included in the analysis. In addition, the temporal relationship between the associated retinal disease and ERM development could not be established retrospectively in all cases; therefore, some ERMs classified as secondary may have represented idiopathic age-related ERMs coexisting with other retinal pathology. A detailed assessment of the foveal vasculature was also not available. Additionally, cataract surgery performed during the follow-up period may have influenced BCVA outcomes, particularly at the 12-month assessment, and its contribution to visual improvement cannot be completely separated from that of ERM surgery. OCT images were evaluated by a single unmasked observer, and intraobserver reproducibility was not formally assessed, which may have introduced assessment bias.

## 5. Conclusions

In conclusion, PPV for ERM resulted in significant functional and anatomical improvement, with improved visual acuity and reduced central macular thickness during follow-up. These findings support the effectiveness of surgical treatment for ERM, although postoperative visual outcomes may be influenced by multiple clinical factors.

## Figures and Tables

**Table 1:** Demographic Characteristics of the Patients.

Characteristic	Value
Number of patients	51
Number of eyes	57
Gender (male/female)	25 (49%)/26 (51%)
Mean age (years)	71.43 ± 6.46
Laterality	Right: 28 (49%) Left: 29 (51%)
Lens status	Phakic: 28 (49%) Pseudophakic: 29 (51%)
ERM etiology	Idiopathic: 34 (59.6%) Secondary: 23 (40.4%) • Diabetic retinopathy: 17 (29.8%) • Age-related macular degeneration: 3 (5.3%) • Retinal vein occlusion: 3 (5.3%)

ERM: Epiretinal membrane.

**Table 2:** Changes in Best-Corrected Visual Acuity (BCVA) Before and After Surgery.

Time Point	Mean BCVA (logMAR) ± SD	*p*-Value (vs. Pre-Op)
Preoperative	0.78 ± 0.35	–
1 month	0.76 ± 0.46	0.625
3 months	0.66 ± 0.43	0.021
6 months	0.69 ± 0.52	0.044
12 months	0.62 ± 0.44	0.004

BCVA, best-corrected visual acuity; SD, standard deviation.

**Table 3:** Changes in central macular thickness (CMT) before and after surgery.

Time Point	Mean CMT (µm) ± SD	*p*-Value (vs. Pre-Op)
Preoperative	411.68 ± 124.13	–
1 month	313.24 ± 127.94	<0.0001
3 months	275.63 ± 107.70	<0.0001
6 months	256.71 ± 100.23	<0.0001
12 months	243.03 ± 89.25	<0.0001

CMT, central macular thickness; SD, standard deviation.

**Table 4:** Comparison of the primary analysis and sensitivity analysis restricted to one eye per patient.

Outcome	Primary Analysis (57 Eyes)	Sensitivity Analysis (51 Eyes)
Preoperative BCVA, logMAR	0.78 ± 0.35	0.79 ± 0.35
Final BCVA, logMAR	0.62 ± 0.44	0.66 ± 0.45
Change in BCVA, logMAR	−0.16 ± 0.46	−0.13 ± 0.49
*p* value	0.004	0.039
Preoperative CMT, µm	411.68 ± 124.13	403.22 ± 124.58
Final CMT, µm	243.03 ± 89.25	243.49 ± 95.27
Change in CMT, µm	−168.64 ± 119.65	−159.73 ± 120.41
*p* value	<0.001	<0.001

BCVA, best-corrected visual acuity; CMT, central macular thickness; logMAR, logarithm of the minimum angle of resolution.

## Data Availability

The data presented in this study are available on request from the corresponding author due to privacy and ethical restrictions.
